# Supplementary material for: Relative age of youth swimmers and their sporting performance at the end of the season
Source: PLoS One. 2025 Oct 28;20(10):e0335041. doi: 10.1371/journal.pone.0335041 (PMC12561930; doi:10.1371/journal.pone.0335041)
Supplement: S1 Table — (DOCX) [file pone.0335041.s001.docx]

Table 1: Reliability analysis.

Scale Reliability Statistics

**Media Alfa de Cronbach ω de McDonald Scale** 3.97 0.761 0.792

Element Reliability Statistics

**If the element is discarded**

|  | **Media** | **DE** | **Alfa de Cronbach** | **ω de McDonald** |
| --- | --- | --- | --- | --- |
| **Coach 1** | 3.88 | 1.053 | 0.744 | 0.769 |
| **Coach 2** | 3.85 | 0.939 | 0.683 | 0.731 |
| **Coach 3** | 4.36 | 0.742 | 0.673 | 0.700 |
| **Coach 4** | 4.12 | 1.083 | 0.782 | 0.804 |
| **Coach 5** | 3.64 | 0.783 | 0.708 | 0.760 |
