# Supplementary material for: Relative age of youth swimmers and their sporting performance at the end of the season
Source: PLoS One. 2025 Oct 28;20(10):e0335041. doi: 10.1371/journal.pone.0335041 (PMC12561930; doi:10.1371/journal.pone.0335041)
Supplement: S2 Table — (DOCX) [file pone.0335041.s002.docx]

**Table 2: Coach perception questionnaire.**

| **COACH** |  | **EVALUATE EACH SWIMMER IN THE PARTICIPATING GROUP FOR EACH REQUIRED ITEM IN ITS DIFFERENT GRADATIONS AND BASED ON THE INDIVIDUAL TECHNICAL CRITERIA OF THE COACH RESPONSIBLE FOR EACH TRAINING GROUP** (RATE FROM 1 TO 5, where **1 = STRONGLY DISAGREE** and **5 = STRONGLY AGREE**) | | | |
| --- | --- | --- | --- | --- | --- |
| **GROUP** |  |  |  |  |  |
| **CATEGORY** |  |  |  |  |  |
| **SWIMMER - NAME** | **1.- Understands the content and explanations provided by the coach during training sessions** | **2.- Performs the tasks as instructed** | **3.- Adjusts behavior and execution based on corrections and feedback from the coach** | **4.- Shows maturity, enabling proper integration into the training group** | **5.- Has tools to compensate for deficits in some training aspects and applies them** |
|  |  |  |  |  |  |
|  |  |  |  |  |  |
|  |  |  |  |  |  |
|  |  |  |  |  |  |
|  |  |  |  |  |  |
|  |  |  |  |  |  |
|  |  |  |  |  |  |
|  |  |  |  |  |  |
|  |  |  |  |  |  |
|  |  |  |  |  |  |
|  |  |  |  |  |  |
|  |  |  |  |  |  |
|  |  |  |  |  |  |
|  |  |  |  |  |  |
