# Supplementary material for: Relative age of youth swimmers and their sporting performance at the end of the season
Source: PLoS One. 2025 Oct 28;20(10):e0335041. doi: 10.1371/journal.pone.0335041 (PMC12561930; doi:10.1371/journal.pone.0335041)
Supplement: Attachment — Submitted filename: Response to Reviewers PLOSOne.docx [file pone.0335041.s004.docx]

**Response to Reviewers – Full Letter**

**We sincerely thank the reviewers for their thoughtful comments and suggestions, which have helped us significantly improve the clarity, methodological rigor, and presentation of our manuscript. Below, we provide detailed, point-by-point responses to each comment.**

**Response to Reviewers**

***Reviewer #1:*** *Overall, the study addresses an important and timely topic—the influence of the relative age effect (RAE) on the athletic and academic performance of young swimmers—using a generally sound methodological approach. The article is also fairly well written, with clear language and a logical structure that makes it accessible to readers. However, the study as a whole comes across as somewhat unfocused and overly broad.
The inclusion of a wide range of variables—from anthropometric data and academic grades to the coach’s subjective evaluation—combined with a relatively small and heterogeneous sample, makes it difficult to draw clear conclusions and somewhat blurs the central message of the article. A more focused design, with fewer but more coherent variables, and a larger, more homogeneous sample—e.g., grouped by distance specialization or age category—would likely result in a clearer and more impactful contribution.
Below, I present specific comments and suggestions related to section of the manuscript.*

**Thank you very much for your comments. We would now like to address some of your observations and specify the changes that have been made to the document accordingly.**

*1) Introduction
Although the introduction provides a broad and comprehensive overview of the literature on RAE in sports, it lacks a clearly defined research gap that would justify this study. The authors cite numerous relevant studies and refer to multiple performance-related factors, but they do not clarify what specifically is missing in the current body of knowledge—especially in the context of swimming as a technical and individual discipline. Furthermore, it remains unclear whether the originality of this research lies in the national setting, the age group studied, or the competitive level analyzed. To strengthen the rationale for the study, the authors should more explicitly define the knowledge gap and explain how their study aims to address it.
Additionally, while the authors do state the objectives of the study at the end of the introduction, they fail to formulate specific, testable hypotheses. Given the quantitative nature of the research and its aim to explore relationships between RAE and multiple performance-related variables, the absence of clearly articulated hypotheses weakens both the methodological clarity and the coherence of the interpretation. The authors are encouraged to clearly state their assumptions and expectations, including the predicted direction and strength of the relationships under investigation.*

**We have revised the introduction to more explicitly state the research gap regarding RAE in swimming within the Spanish context. Additionally, we now include clear, directional hypotheses based on the reviewed literature.**

*2) Participants
While the sample includes national-level swimmers, the age range (mean age 15 ± 2.08 years) introduces substantial variability that may impact the interpretation of the results. In adolescent athletes, particularly around the age of 15, even one year can correspond to significant differences in biological maturity, anthropometric characteristics, and physical performance. This variation becomes even more pronounced when comparing athletes with different specializations, such as sprinters versus long-distance swimmers, who differ considerably in their developmental and physiological profiles. The presence of athletes with an age difference of more than two years within the same sample means that the study is comparing individuals at very different stages of physical and psychological development and sport specialization, which could limit the validity of the conclusions drawn.*

**The differentiation that may appear among adolescent athletes is well known, and this degree of variability is generally acknowledged. The aim of this study was to establish potential relationships between each swimmer’s performance and their category standard, considering the changing conditions and demands associated with category transitions. For this reason, it is stated that athletic performance is assessed based on specific results within each competitive category, placing athletes within the same performance ranges while considering the adjustments in qualifying standards and access to various levels.**

**Regarding the differentiation by stroke specialties and preferred swimming distances, these factors were not considered in the analysis due to the very reasons mentioned: the limited sample size and the added complexity such analysis would entail. Only part of this information is included as supplementary data (this was suggested by another reviewer).**

*3) Discussion
The discussion begins by restating the main objectives of the study in terms that closely mirror the introduction. While briefly revisiting the aims is acceptable, this repetition adds little value unless it is immediately tied to the study’s findings. It is recommended that the authors revise the opening to more directly present and interpret their key results in light of the study’s goals.
The discussion covers a wide range of topics and successfully links the study's findings to existing research, addressing both statistically significant and non-significant outcomes. The authors appropriately acknowledge several limitations, such as the small sample size and the imbalance in gender and specialization. However, at times the section feels overly long and resembles a literature review, which can dilute the focus on the study’s own results.
It is commendable that the authors attempt to consider the combined influence of social, anthropometric, and psychological factors. Nevertheless, some of their interpretations are speculative and not clearly supported by their own data—for instance, references to the Pygmalion effect or the influence of coaching style. A more distinct separation between data-driven conclusions and those based purely on theoretical or literature-derived explanations would improve clarity.
Furthermore, the discussion would benefit from a more concise and explicit presentation of the practical implications of the findings, especially for coaches, sport program designers, and federations. Suggested directions for future research are relevant, particularly those that call for the inclusion of measures like biological maturity to better control for individual differences in development.*

**We have considerably shortened and focused the discussion, distinguishing between conclusions based on data and interpretations based on literature. Conversely, we have removed some assessments relating to the athlete's external perception.**

*4) Conclusion
The conclusion could be more concise and avoid repeating results already discussed earlier in the manuscript. It would also benefit from a clearer focus on the practical implications for coaches, training programs, and youth sport planning.
The authors’ suggestions for future research are a strength of the section. Highlighting the need for larger samples and comparative studies in other sports and age groups enhances the broader applicability of the findings. However, these more detailed research proposals might be better placed in the final part of the discussion, rather than in the conclusion itself, which should ideally focus on the key takeaways from the study.*

**We revised the conclusion to emphasize applied implications for coaches and removed redundancies present in earlier sections. Aspects aimed at future research directions and the practical utility of this information have also been included at the end of the discussion section.**

**Reviewer #2**:

*Dear Authors,

I want to express my gratitude for the opportunity to review this manuscript.

Congratulations on the study. Some improvements are suggested below, with line indications.

L69-70 – Please consider abbreviating “PA”.*

**The changes have been made by incorporating the acronyms so they can be included in the rest of the text.**

*L98 – “relative age” – RAE suggested.*

**The changes have been made by incorporating the acronyms so they can be included in the rest of the text.**

*L108 – Please describe the inclusion and exclusion criteria. Please describe all available information related to the subjects´ characterization. Some examples include training routines, years of experience, competitive level, and number of weekly training sessions.*

**The selection, inclusion, and exclusion criteria are described in the second paragraph of the Design and Procedure section.**

*L121-188 - Please describe all methodological details, for example, the procedures in detail, preferably with reference support. Another example is the human resources involved – academic background and experience.*

**The text has been slightly modified, and a related reference has been incorporated into the opening paragraph of the Instruments section. In the Design and Procedure section, additional details have been included regarding the process and the research team** *L191-206 – Please consider shorter paragraphs to improve readability (8-12 lines suggested). Please make sure all statistical procedures are described.*

**The text has been revised and refined to enhance clarity and coherence in the discourse adding more information about procedures.**

*L216 – Please revise all tables´ content and format, considering the journal template and instructions for authors.*

*L207-282 – The reformulation of this section is suggested to become more appealing and easier for readers' interpretation. Please revise the text and tables.*

**The text has been revised and refined to enhance clarity and coherence in the discourse adding more information about procedures.**

*L286 – Only “RAE” suggested, previously in full. Please revise all manuscript.*

**The term has been replaced by its acronym throughout the different sections of the text.**
 *L290-420 - Please consider improving the quality of the discussion section, namely regarding the study rationale, and with the inclusion of more references.*

**We have significantly adapted and focused the text, distinguishing between conclusions based on the data and interpretations based on the literature.** *L420 – Please consider indicating the study limitations and suggestions for future research,
L422 - Please consider short and clear take-home messages, if possible, with practical applications/implications.*

**We revised the conclusion to emphasize applied implications for coaches and removed redundancies present in earlier sections. Aspects aimed at future research directions and the practical utility of this information have also been included at the end of the discussion section.** *L456 - Please double-check the references format. For example: Titles in upper and lowercase; Journal in full and abbreviated.*

**The text has been revised in accordance with the journal's guidelines.**

**Reviewer #3:**

*General Feedback
The manuscript presents valuable original research on the relative age effect (RAE) in youth swimmers. The study research question is highly relevant, and the methodology is sound, highlighting how the RAE influences performance conditionally, which is a significant contribution. However, the paper can be strengthened by addressing some areas in different sections as suggested.*

*Abstract
The abstract can be made more concise while still including all essential information, especially about your novel findings (e.g., direct versus mediated effects).*

**A more precise revision of the abstract has been completed**

*Introduction
• Given your extensive literature review, are there any specific contradictory findings in previous research that your study directly aims to clarify or challenge?*

**Thank you for your comment. No contradictory studies on the subject of research on swimmers have been found. We wanted to test the hypothesis put forward at the end of the introduction against the sample to see if it holds true and confirms the previous findings.**

*• The introduction could benefit from a clearer articulation of the specific gap in the literature that this study directly aims to fill, beyond generally exploring RAE in swimming.*

**We have revised the introduction to more explicitly state the research gap regarding RAE in swimming within the Spanish context.**

*• The topic and idea are valid. However, the article reads more like a description than a true scientific analysis guided by a clearly defined theoretical framework. The theoretical framework connecting RAE to swimming performance requires enhancement, and it is important to include a comparison with similar studies on RAE.*

**Thank you for your comment. We have reviewed the introduction, making changes according to your suggestions.**

*Figures and tables
Could a graphical representation of key findings, such as the relationship between birth quartile and performance when mediated by BMI, enhance readers' understanding of your results?*

**New tables have been added**

*Methods
• Could you elaborate on your rationale for using a convenience sample and discuss any potential biases this might introduce?
• Given the acknowledged sample size limitation; (a). what steps did you take during the analysis to account for this (e.g., sensitivity analyses)? (b) Did you conduct any power analysis to determine the necessary sample size to detect expected effects?*

**A note was added in the text acknowledging the lack of a priori power analysis and the exploratory nature of the study due to sample access constraints.**

*• You have given 5 items on the "coach's subjective perception" based on ad hoc questionnaire. Could you provide a copy of the full questionnaire as supplementary material for reproducibility?*

**A copy of the ad hoc questionnaire template provided to the coaching staff is enclosed as a supporting document. It is important to note that the information gathered through this instrument was incorporated as supplementary data to the athletic performance outcomes, serving as an interview-based account and offering each coach’s subjective perspective on their respective group.**

*• The specific procedures for data collection should be further explained. For example, how were the questionnaires administered, what were the exact anthropometric measurement protocols if they deviate from standard including reference?
• Are there any other specific procedural details that you could expand upon for greater transparency and reproducibility? e.g., order of measurements, instructions given to swimmers during anthropometry etc.*

**The text has been slightly modified, and a related reference has been incorporated into the opening paragraph of the Instruments section. In the Design and Procedure section, additional details have been included regarding the process and the research team.**

*Statistical Analysis and Result
• Could you present a more detailed breakdown/post hoc analysis of the significant differences in ANCOVA. For instance, could you clarify which quartiles demonstrated significant differences, such as between Q1 and Q4, regarding the means and standard deviations of the dependent variables?
• The ANCOVA results are presented with F-values and p-values, but not the specific group means for the factors involved. Could you provide the means and standard deviations for the dependent variables across different/individual quartiles or months of birth, and training days, to better illustrate the significant differences?
• The specific statistical approach such as Tukey's HSD, FDR etc., to address the issue of multiple comparisons given the number of correlations and ANCOVA analyses performed are not explicitly stated. This represents a limitation in your statistical methodology, as multiple comparison corrections would have strengthened the reliability of your findings.*

**New writing has been added.**

**As suggested, we included post hoc comparisons following ANCOVA and provided tables with means and standard deviations by birth quartile and month. We clarified in the data analysis section that Tukey HSD corrections were used to address multiple comparisons where applicable.**

**Post hoc comparisons using Tukey’s HSD test indicated statistically significant differences in sport performance between swimmers in Q4 and those in Q1, Q2, and Q3. The greatest difference was observed between Q2 and Q4 (*p* = .01), suggesting a potential relative age disadvantage for athletes born in the last quartile of the year.**

*Discussion
• Could the authors expand on how their findings, particularly the conditional influence of RAE, challenge or refine existing theories on RAE in sports?*

**Thank you for your comment. This section was realigned in line with the recommendations of other reviewers.**

*• Given the stated limitations in the methods, what specific future research directions are most crucial to build upon these findings?*

**We revised the conclusion to emphasize applied implications for coaches and removed redundancies present in earlier sections. Aspects aimed at future research directions and the practical utility of this information have also been included at the end of the discussion section.**

*• How might the acknowledged gender imbalance in the sample affect the generalizability of the findings to male swimmers, even if no gender differentiation was observed within the study?*

**Thank you for your comment. We understand that no gender differentiation was made within the study, as there were no conditions for comparison in the sample. However, a possible tendency for positive performance differentiation in favour of the female gender was observed in the literature.**

*• Could you expound on how these findings compare to RAE studies in other individual sports?*

**Thank you for your comment. No comparisons with other sports were made within our analysis, as we wanted to focus this section on swimming. However, other individual sports are alluded to in reference [43], which is based on triathlon and analyses sports such as swimming, cycling and athletics. This reference provides results showing how the RAE influences sporting performance.**

*Conclusions
• Could the practical implications of the findings be more explicitly stated for coaches, parents, and sports organizations?
• How might the "global plan" mentioned in the conclusions be structured or implemented based on these findings?*

**The conclusion section was revised to highlight the practical implications for coaches, while redundant content from earlier sections was removed. Additionally, the final part of the discussion now includes considerations for future research and emphasizes the practical relevance of the findings.**

*Additional aspects
• It is stated in the manuscript that, "All data are fully available without restriction," implying deposition, but you haven't provided specific repository or accession numbers for the data. This is crucial for transparency and reproducibility as per the journal guidelines.*

**Attached is the study's database, which shows the sample data and measured variables. This file can be found under the name 'Database Swimmers.xlsx'.**

*• STROBE (Strengthening the Reporting of Observational Studies in Epidemiology) would be relevant guidelines for this work. Please ensure full adherence.*

**Standard research protocols and guidelines have been followed regarding the procedures to be undertaken in studies of this nature.**
