# Supplementary material for: Relative age of youth swimmers and their sporting performance at the end of the season
Source: PLoS One. 2025 Oct 28;20(10):e0335041. doi: 10.1371/journal.pone.0335041 (PMC12561930; doi:10.1371/journal.pone.0335041)
Supplement: Attachment — Submitted filename: Response to Reviewers.docx [file pone.0335041.s005.docx]

**Response Letter to the Reviewers – Manuscript “Relative age of youth swimmers and their sporting performance at the end of the season”**

Dear Dr. Mohamed Ahmed Said,

Academic Editor of PLOS ONE,

Dear Reviewers,

We deeply appreciate your valuable comments and the time you have dedicated to reviewing our manuscript. We are grateful for your acknowledgment in your recent communication that we have corrected, modified, and incorporated nearly all of the suggestions and improvement recommendations provided by the reviewers in the previous round.

Below, we present a new point-by-point response to each of the comments received, indicating the new modifications made to the previously revised version of the manuscript and justifying those that could not be implemented. Modifications and changes in citation numbering within the text have been highlighted in yellow. New references have been added to the corresponding section and are also highlighted in yellow.

We hope this clarifies the points that remained pending in the reviewers’ judgment.

**Reviewer 1**

Comment: It is suggested to incorporate contradictory or diverse findings from the literature to enrich the impact of the study.

Response: We have expanded the literature review previously modified, including studies on the Relative Age Effect (RAE) in other individual sports such as athletics, tennis, taekwondo, and gymnastics, where divergent results have been observed (lines 371–377; 385–389; 408–410; 425–428; 430–436). This contextualization has been added to the introduction and discussion sections to strengthen the interpretation of our findings in swimming. The new references have been added to the final section and are highlighted in yellow.

**Reviewer 2**

Comment: Additional information is required regarding the human resources involved, including academic background and experience of the evaluators.

Response: A detailed description of the academic profile and professional experience of the evaluators was already included in the methodology section of the previous version. In this version, we have expanded it with additional data (lines 219–226).

Comment: Some paragraphs in the results section are lengthy.

Response: We have further segmented the section into shorter and more distinct paragraphs to improve readability and facilitate understanding, as recommended. Additionally, in previous versions of this manuscript, a reviewer had suggested expanding certain explanations, which we did in accordance with that recommendation.

**Reviewer 3**

Comment: Justification for convenience sampling.

Response: We have expanded the justification for convenience sampling in the methodology section, explaining its inherent limitations and potential impact on the generalizability of the results (lines 127–137).

Comment: No power analysis was conducted.

Response: We have added an explicit statement in the limitations section indicating that no power analysis was conducted due to the exploratory nature of the study and the sample size (lines 476–481). Reliability evidence is provided in the instruments section.

Comment: The coach perception questionnaire lacks evidence of validity and reliability.

Response: We have acknowledged this limitation in the methodology/instruments and discussion sections, indicating that the instrument has not been formally validated and that the data obtained should be considered exploratory. Additionally, we have added a note on the need for future studies to validate this questionnaire. Reliability evidence has been added (Cronbach’s Alpha and McDonald’s ω, lines 180–185). These data will be included as a supplementary document for reviewer consultation in the journal’s annexes.

Comment: Limited discussion on similarities with other individual sports.

Response: We have expanded the discussion to include references to studies on RAE in sports such as judo, taekwondo, gymnastics, and athletics, to enrich the interpretation of the results—both in terms of confirming the RAE and studies indicating that some results are not definitive (lines 371–377; 385–389; 408–410; 425–428; 430–436). The new references have been added to the final section and are highlighted in yellow.

Comment: Confirm that the supplementary dataset complies with the journal’s policy.

Response: We confirm that the dataset related to this work has been provided to the journal for reviewer and editor consultation, ensuring access and availability should it be required.

**Cross-cutting Issue: Validity and Reliability of the Questionnaire**

Comment: The questionnaire has not been validated.

Response: We have added an explicit statement in the methodology/instruments and discussion sections acknowledging that the questionnaire has not been validated in previous studies. However, it has undergone reliability testing (Cronbach’s Alpha and McDonald’s ω). These data have been incorporated into the text (lines 180–185; 476–481; 512–517). The data will be included as a supplementary document for reviewer consultation if desired. We indicate that the data obtained are exploratory and suggest conducting future studies to validate the instrument. The limited sample size restricts the ability to detect stronger internal consistency, but this limitation has been noted as a methodological constraint.

We reiterate our gratitude for your observations, which have significantly contributed to improving the quality and rigor of our work. We remain attentive to any further suggestions you may consider appropriate.

Sincerely,

Dr. Daniel Mendoza Castejón, on behalf of the research team.
